# Nursing Institutions and Hospitals

**Published:** 1887-03-05

**Authors:** E. A. Bedingfeld


### NURSING INSTITUTIONS AND HOSPITALS.

### THE CASE FOR THE PRIVATE INSTITUTIONS AND NURSES.

MRS. E. A. BEDINGFELD,
Lady Superintendent, the Belgravia Institution for Trained Nurses, 25, Lupus Street, S.W.

WILL you allow me to say a few words regarding Miss Manson's article on Nursing, etc., in THE HOSPITAL of Feb. 19, in the interests of both the private nursing institutions, and the nurses employed in and by them, as some of her remarks are not only incorrect, but unfair? First, as to Miss Manson's statement that the institutions "farm the nurses out for their own profit." Is not this precisely what the hospitals intend doing? Then, nurses must have a home, and surely it is better for them to enter an institution in which, in addition to a fair salary, they have, when they return from their work, the comforts of a home waiting them, without any trouble on their part, than to live in lodgings alone. There is neither evil nor injustice in this. It is simply mutual accommodation. Again, it is *not* correct that some of the nursing institutions "engage women who have had no previous experience of nursing," unless it is as "probationers," in which case they are sent to a hospital to be trained. It surely matters little whether a woman enters a hospital on her own account, or is sent by an institution. The training given by the hospital ought to be the same in either case; and if it is not, it is the fault of the hospital for not refusing to train anyone unless they stay long enough to become competent. Nursing institutions are, to my positive knowledge, very particular as a rule, in inquiring into the character and antecedents of women who represent themselves as fully trained. I, for one Superintendent, always insist on references given direct to myself, either personally or by letter, and require that a nurse should have had *at least* one year's training. The properly trained nurse has her hospital certificate to show; and if the public are not to believe that, it does not say much for the conscientiousness of those who fill in and sign it.

As a rule, nurses are not "found" in institutions, unless, as in the case of one or two, uniform is provided. The statement, that "a highly competent nurse rarely enters an institution," is absolutely incorrect. I may say that a nurse's training is *far* from complete in many ways, when she leaves the hospital. So far from it, that many Superintendents, myself amongst the number, strongly object to take a nurse on our staff direct from the hospital, because, except in rare cases, though she may be perfect in her actual work, she has no knowledge of the niceties and refinements of the private sick-room. Her rule is too stern. She is used to the routine and implicit obedience which obtain in hospitals, but which *cannot* always be enforced in private work, and she lacks usually the tact requisite for the management of those who, when well, are a law unto themselves. All this she has to learn. Again, a nurse has often to act as lady's-maid, if not as valet. Invalids require their hair brushed and toilette attended to, in sickness as well as in health, and these duties are certainly not taught in hospitals. I have had it said to me, "Don't give me a nurse fresh from her training I don't wish to be *experimented upon*."

Further, does Miss Manson mean that no nurses are to be employed but those in the private homes attached to the hospitals? She says that competent nurses rarely enter a private institution, and that hospitals retain their best nurses for themselves; *ergo*, there are no *good* nurses to be got outside the hospital homes. If the hospitals retain all their good nurses, where will, by-and-bye, be the room for new relays to be trained, as the hospitals and homes will be overcrowded? And what is to become of those numbers who, up to the present time, have left the hospitals after their period of training is over, holding certificates of *thorough* training, but who do not care to remain on in the hospital? Are they to starve because the hospitals, in order to increase *their* incomes (for this is the *real* reason why the hospitals are sending out private nurses, and they would be better thought of if they honestly said so), are striving to take the bread out of the mouths of others? In more than one instance a hospital has so reduced its own staff by sending out to private cases, that they have had to get in "specials" from where they could to do the hospital work. "Live and let live," I say. There are now more good nurses in London than there is work for. I have known instances this winter of nurses, holding first-class certificates, who have, through no fault of their own, been almost starving for want of work; and if what Miss Manson proposes be carried into effect, their number will be largely increased.

One word as to uniform. In very many instances it is strongly objected to, and I have had said by both doctors and patients, "I hope your nurses do not wear uniform." The apron is even sometimes, though rarely, an offence; but the cap often is. Some think it makes the nurse look too much like the servants. Others, in mental cases especially, when the nurse has to be so much in the drawing-room, and to go out with the patient, like the nurse to look as *unlike* a nurse as possible. So, though the cap and apron are desirable, the wearing them cannot always be enforced. All nursing institutions (and they are many) with which I am acquainted, send rules with their nurses, framed in the interest of both nurse and patient—I enclose a copy of mine—and nurses are required to bring back a statement of their conduct while at the case. I think Miss Manson can have had but little practical experience of nursing institutions, or she would not make the assertions she has done. I have been Superintendent of one for some years, and have been interested in learning the internal working of others, so perhaps my words may have a little weight; and as, to have a proposition fairly considered, it must be looked at from all points of view, may I trust to your courtesy to give expression to my views, which I may say are shared by several nurses to whom I have shown them?

MISS MEYRICK,
Lady Superintendent of the Hospital for Gentlewomen, Harley Street.

I WAS unfortunately prevented from being in time to hear Miss Manson's paper read on "Is it desirable for Metropolitan Hospitals to possess Nursing Institutions, etc.," and I could not therefore, even if I had had the courage, accept the chairman's invitation to join in the discussion upon it. Cordially as I agree with almost the whole of the very interesting paper, there is one point upon which I should like to say a few words. I gather that Miss Manson wishes the nurses employed outside the hospital walls, to wear an outdoor, as well as an indoor uniform. The indoor uniform I think quite as important in every way as she does, but I differ from her about the outdoor uniform, and for this reason: I think it is not only natural, but right, that women should try to make themselves look as well as they can, and it seems rather hard that young women—and it is young women that we specially desire for nurses—should be obliged to forego the innocent pleasure that a pretty dress or bonnet gives, just when they most want the change from sick-room surroundings.

Nurses do not generally take up their work, as women do who go into the religious life, giving up all thoughts of earthly happiness or advancement. If an earnest young woman really desires to give up the world and worldly dress, the chances are that she will join a nursing sisterhood. I cannot help thinking that the outdoor uniform is one reason why so many nurses leave the hospital after their three years' training. Naturally, nurses make little objection to wearing outdoor uniform during these three years, as that, or anything else, is but a small price to pay for the teaching which puts so noble a profession into their hands. But one gets tired of the prettiest style of dress after a time, and wants a change, even when that style is of one's own choosing,—and some of the outdoor uniforms are, I must say, very ugly. In talking on this subject some time ago, I was told that the outdoor uniform was so very useful, as the hospital under discussion was situated in such a low neighbourhood, it prevented the nurses from going to undesirable places, or behaving themselves in an undesirable manner. Surely women who would, in any dress, go to such places, are not the women we desire for nurses, to whose care and tenderness we trust the sick, the helpless, and the dying. A good woman, who is also a good nurse, can surely be trusted to dress in a manner suited to the dignity of her calling. It is these women who have the least to fear in cutting themselves off from the hospital, and starting on their own account; but it is also these women whose services it is specially desirable to retain. I think it would not be unworthy of a small place in the training the Matron gives her probationers if, instead of the outdoor uniform, she tried to show them that the style of dress is an index of the mind of the person, and, while encouraging them to dress becomingly, she endeavoured to teach them that a modest, suitable dress would most conduce to get themselves and their calling duly respected. In nursing mental cases too, where the nurse has to go out with the patient, the uniform may often be objectionable, though, of course, a special permission could always be given not to wear it. I think that the hospital nursing homes will get better nurses, and retain them longer, if an outdoor uniform is not insisted on.

[Miss Meyrick seems to overlook the fact that if a nurse is compelled to go into low courts and alleys to visit the sick, her uniform would show the object of her presence, and so constitute a more efficient protection than the conduct of a policeman.—Ed. T. H.]

### A Trained Nurse's View.

In justice to many women engaged by private institutions to nurse the rich and middle classes, will you allow me to try to remove the impressions which such assertions as Miss Manson makes in her paper published in The Hospital of February 19th are likely to create? To say that "it is only now and again that a highly competent nurse enters a private institution," is untrue. The success of such institutions depends entirely on the abilities and competence of the nurses they send out; and those who do supply incompetent women must find it, in the long run, against their own interests. It would be impracticable for all good nurses to remain attached to the hospitals where they have trained, as after a time provincial hospitals could not find work for them.

It is incorrect to say that a well-trained nurse can become "rusty," for an observant woman will always be able to perform her work in the sick-room and at the operating-table. She will be willing to follow out the doctor's orders, and if she does that faithfully she cannot go far wrong. Miss Manson says that the need of sick-nurses is met by "private nursing-homes, in the hands of private individuals, who farm out nurses for their own profit." She might with justice have stated that in private nursing-homes connected with hospitals the nurses' salaries will bear no comparison with their earnings. At one of the private institutions, however, started by a noble woman to remedy this injustice, the nurses receive their own earnings.

I was for nine years at the training school and private nursing home of one of the best of our provincial hospitals, to which a medical school is attached. When I left to try to get more benefit from my work, my salary was £28 per annum. Had I remained another nine years it would only have been £30. I think the most right-minded persons will consider that it is no more just to divert the profits of a nurse's work to the funds of a hospital, than into the hands of private individuals, who have the risk and expenses connected with the working of their establishments.

Lastly, I fail to see what outdoor uniform can have to do with a private nurse's capabilities. There is no good reason why nurses should be advertised in the streets, and many families object to those they employ going out of their houses in a conspicuous dress. I have written this letter on the principle of "live and let live"; for such sweeping assertions regarding women who have many of them given their best days to hospital and private work, cannot fail to leave a sore feeling against those who make them.

### FREE HELP IN SICKNESS.

A voluntary hospital is an ever-open home, where the hosts are boundless love and meek and lowly charity. These are always eager to extend their everlasting hospitality to the sick, the halt, the maimed, and the blind. Here the poor in wealth and the poor in spirit are welcomed by Christian hearts, and received by Christian hands.—Condorcet.

